# Inducible SMARCAL1 knockdown in iPSC reveals a link between replication stress and altered expression of master differentiation genes

**DOI:** 10.1242/dmm.039487

**Published:** 2019-10-01

**Authors:** Giusj Monia Pugliese, Federico Salaris, Valentina Palermo, Veronica Marabitti, Nicolò Morina, Alessandro Rosa, Annapaola Franchitto, Pietro Pichierri

**Affiliations:** 1 Mechanisms, Biomarkers and Models Unit, Department of Environment and Health, Istituto Superiore di Sanità, Viale Regina Elena 299, 00161 Rome, Italy; 2 Center for Life Nano Science, Istituto Italiano di Tecnologia, Viale Regina Elena 291, 00161 Rome, Italy; 3 Department of Biology and Biotechnology Charles Darwin, Sapienza University of Rome, P.le A. Moro 5, 00185 Rome, Italy; 4 Istituto Nazionale Biostrutture e Biosistemi, Via delle Medaglie d'Oro, 00136 Rome, Italy

**Keywords:** DNA damage, DNA replication, Replication stress, SIOD, IPSC

## Abstract

Schimke immuno-osseous dysplasia is an autosomal recessive genetic osteochondrodysplasia characterized by dysmorphism, spondyloepiphyseal dysplasia, nephrotic syndrome and frequently T cell immunodeficiency. Several hypotheses have been proposed to explain the pathophysiology of the disease; however, the mechanism by which *SMARCAL1* mutations cause the syndrome is elusive. Here, we generated a conditional SMARCAL1 knockdown model in induced pluripotent stem cells (iPSCs) to mimic conditions associated with the severe form the disease. Using multiple cellular endpoints, we characterized this model for the presence of phenotypes linked to the replication caretaker role of SMARCAL1. Our data show that conditional knockdown of SMARCAL1 in human iPSCs induces replication-dependent and chronic accumulation of DNA damage triggering the DNA damage response. Furthermore, they indicate that accumulation of DNA damage and activation of the DNA damage response correlates with increased levels of R-loops and replication-transcription interference. Finally, we provide evidence that SMARCAL1-deficient iPSCs maintain active DNA damage response beyond differentiation, possibly contributing to the observed altered expression of a subset of germ layer-specific master genes. Confirming the relevance of SMARCAL1 loss for the observed phenotypes, they are prevented or rescued after re-expression of wild-type SMARCAL1 in our iPSC model. In conclusion, our conditional SMARCAL1 knockdown model in iPSCs may represent a powerful model when studying pathogenetic mechanisms of severe Schimke immuno-osseous dysplasia.

## Abstract (precis)

**Summary:** This study reports the generation of an iPSC model to investigate phenotypic changes associated with loss of SMARCAL1, as found in severe forms of the genetic disease Schimke immuno-osseous dysplasia.

## INTRODUCTION

Schimke immuno-osseous dysplasia (SIOD) is an autosomal recessive genetic osteochondrodysplasia characterized by dysmorphism, spondyloepiphyseal dysplasia, nephrotic syndrome and frequently T cell immunodeficiency (Boerkoel et al., 2000; Clewing et al., 2007; Saraiva et al., 1999). Patients usually suffer from other less penetrant features and, depending on the severity of the disease, they can undergo premature death in childhood or early adolescence (Clewing et al., 2007). The disease is caused by bi-allelic mutations in the *SMARCAL1* gene (Boerkoel et al., 2002). Although *SMARCAL1* encodes for a protein homologous to the SNF2 family of chromatin remodelling factors and SMARCAL1 has been involved in transcriptional regulation (Patne et al., 2017; Sethy et al., 2018; Sharma et al., 2016), recent works proved that SMARCAL1 is critical during processing of DNA structures at replication forks to promote formation of replication intermediates through its ATP-driven strand-annealing activity (Bansbach et al., 2009; Ciccia et al., 2009).

Based on the pathophysiology of the disease, several hypotheses have been proposed (Boerkoel et al., 2000; Elizondo et al., 2006); however, the mechanism by which *SMARCAL1* mutations cause SIOD are completely unknown. The recent demonstration that SMARCAL1 is crucial in response to perturbed replication, and that recovery from replication stress is hampered by its loss or impaired activity, challenged the canon for SIOD molecular pathology from transcriptional regulation to DNA damage prevention. Thus, it is tempting to speculate that SIOD phenotypes are linked to impaired proliferation or development that could follow the accumulation of DNA damage, similar to what has been proposed for other genetic conditions caused by loss of genome caretaker proteins (Ciccia and Elledge, 2010).

Many mutations in the *SMARCAL1* gene have been identified, ranging from frameshift and deletions, which generally lead to protein loss, to missense mutations that differently affect expression, activity, stability and localization of the protein (Boerkoel et al., 2000; Elizondo et al., 2009). Interestingly, SIOD patients bearing distinct *SMARCAL1* mutations show a different degree of disease severity (Elizondo et al., 2009). Thus, a phenotype-genotype correlation might exist, although it is difficult to ascertain. Indeed, mutations resulting in the almost complete loss of protein are associated with severe SIOD. By contrast, mutations that similarly affect SMARCAL1 ATPase activity give raise to both severe and mild SIOD, arguing for the existence of genetic factors that can modulate disease phenotypes or of additional ATPase-independent SMARCAL1 functions that are affected by missense mutations (Baradaran-Heravi et al., 2012; Elizondo et al., 2006, 2009).

Unfortunately, deletion of *SMARCAL1* in mice or fruit flies fails to fully recapitulate the SIOD disease phenotype (Baradaran-Heravi et al., 2012). Only a study from zebrafish evidenced cell proliferation and developmental defects upon deletion of the *smarcal1* orthologue (Huang et al., 2010), suggesting that loss of SMARCAL1 could affect proliferation and development in humans too. Thus, although likely to exist, the correlation between *SMARCAL1* mutations, replication stress, DNA damage formation, defects in proliferation and impaired development in SIOD pathogenesis is as yet unexplored, largely because of the inability of SMARCAL1 loss to induce all SIOD phenotypes in the existing models of the disease.

Induced pluripotent stem cells (iPSCs) are useful when studying the very first stages of development. Such a model system, although unable to give a systemic view, is very useful for the identification of early events associated with disease pathophysiology. Moreover, it is genetically amenable and can be used to provide cell types for drug screening.

Here, we generated iPSCs in which expression of SMARCAL1 could be downregulated through a Tet-ON-regulated RNAi system to model severe SIOD. Using this cell model, we demonstrated that depletion of SMARCAL1 resulted in reduced proliferation, accumulation of DNA damage, replication defects and DNA damage response (DDR) overactivation. Moreover, our data show that the most striking phenotypes are correlated with increased R-loop accumulation and can be reversed, preventing replication-transcription interference. Most importantly, using our iPSC cell model of severe SIOD, we established that replication-related DNA damage also persists in differentiated cells and that loss of SMARCAL1 affects expression of a subset of germ layer-specific marker genes.

## RESULTS

### Generation and characterization of inducible SMARCAL1 knockdown iPSCs

**Fig. 1.**
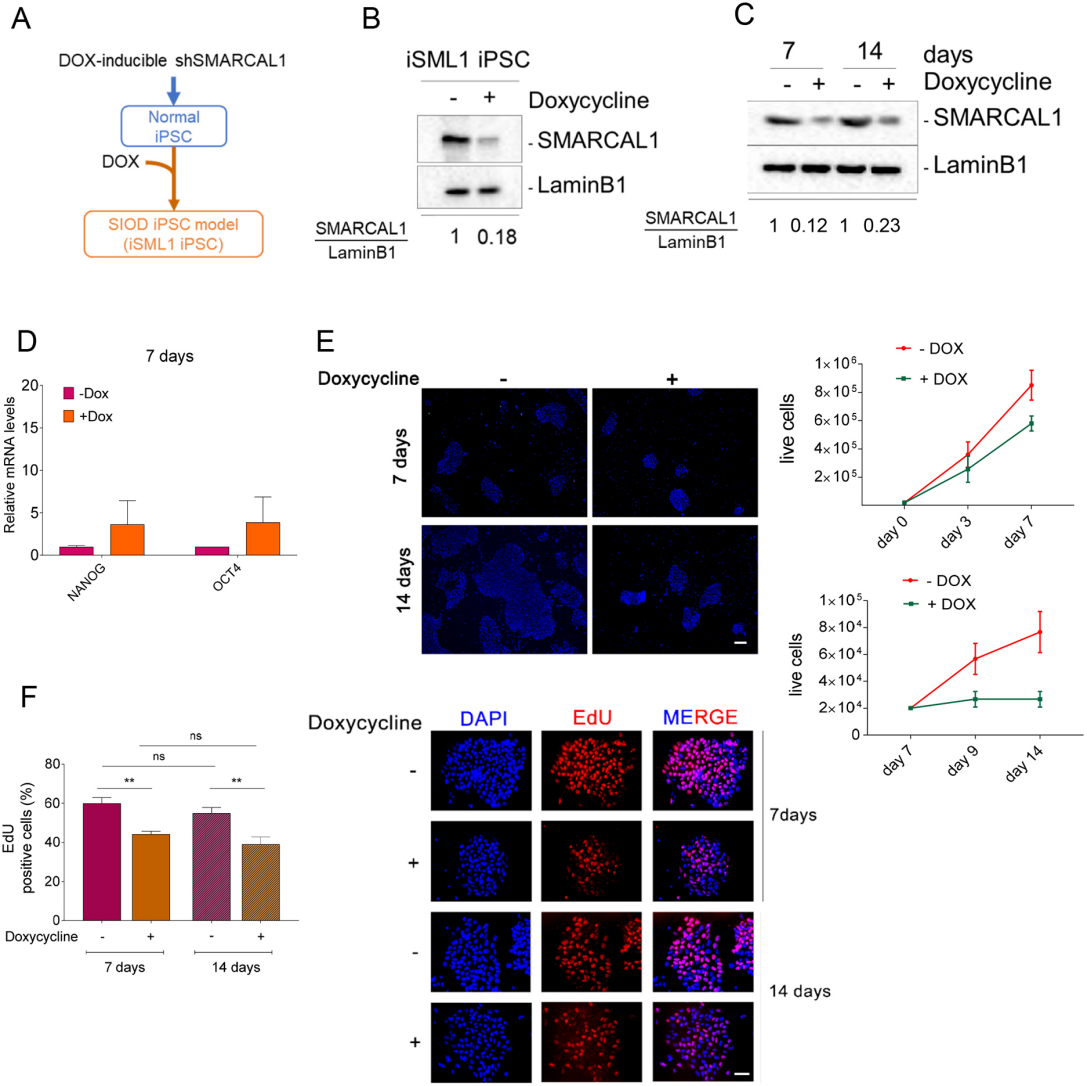
**Generation and characterization of inducible SMARCAL1 knockdown iPSCs.** (A) Schematic representation of the experimental models. iPSCs previously derived from human fibroblasts were infected with the inducible RNAi lentivirus. When challenged with 1 µg/ml doxycycline (DOX) for 7 days, cells were considered SIOD. iPSCs were generated in the absence of DOX and, once established, shifted to a DOX+ media. (B) Western blot showing the efficient silencing of SMARCAL1 after 48 h treatment with DOX. Lamin B1 was used as loading control. (C) Western blot showing the long-term downregulation of SMARCAL1 in DOX-supplemented medium. Lamin B1 was used as loading control. (D) Comparative analysis by qRT-PCR of the expression of pluripotency markers NANOG and OCT4 after 7 days of DOX. Untreated iPSCs were used as reference sample. (E) Analysis of iPSC proliferation. Micrographs (left) show different size of DAPI-stained colonies (blue) from iPSCs cultured in the presence or absence of DOX for the indicated time. Images are representative of different fields. The graphs (right) show quantification of the number of live cells in each population evaluated at the indicated time-points. Student's *t*-test was used for statistical analyses. (F) Analysis of replicating cells in SMARCAL1-depleted iPSCs. Replicating cells were labelled with EdU for 30 min and the graph (left) shows the percentage of positive cells (S-phase) after shSMARCAL1 induction. Representative images (right) show EdU-positive cells (red), with nuclear DNA counterstained by DAPI (blue). Data are mean±s.e.m. from three independent experiments. **P≤0.01 (two-way ANOVA test). ns, not significant (*P*>0.05). Scale bars: 20 µm.

To obtain an inducible model of severe SIOD, we expressed an shSMARCAL1 cassette under the control of a Tet-ON promoter through lentiviral transduction in the well-characterized normal iPSC line WT I (Lenzi et al., 2015) (Fig. 1A). Low-passage iPSCs were infected with the Tet-ON-shSMARCAL1 virus at 0.5 of multiplicity of infection (MOI) by spinfection, selected and tested for the knockdown efficiency by Western blotting. As shown in Fig. 1B, culture of inducible SMARCAL1 protein knockdown (iSML1) iPSCs with doxycycline (DOX) for 48 h resulted in less than 20% of total SMARCAL1. Western blotting analysis of the SMARCAL1 level after 7 or 14 days of continuing growth in DOX revealed that the high knockdown efficiency was stable over time in the iSML1 iPSCs (Fig. 1C).

As the goal of an iPSC model is to generate multiple differentiated cell types, we next analyzed whether SMARCAL1 knockdown altered the expression of pluripotency genes. To this end, cells grown for 7 days in the presence or absence of DOX were analyzed for the expression levels of two key pluripotency genes [*NANOG* and *OCT4* (also known as *POU5F1*)] by real-time (RT)-PCR. The analysis of gene expression showed that SMARCAL1 knockdown does not reduce the expression of the main pluripotency marker genes (Fig. 1D).

Having demonstrated that continuous culturing in DOX-containing medium is effective in maintaining downregulated SMARCAL1, we analyzed whether depletion of SMARCAL1 affected proliferation in iSML1 iPSCs. To this end, iSML1 iPSCs were grown in the presence or absence of DOX for up to 7 or from 7 to 14 days and the number of live cells recorded over time. Although SMARCAL1 downregulation had little effect on proliferation of iSML1 iPSCs during the first week after shSMARCAL1 induction (Fig. 1E), it greatly impaired proliferation from 7 days of growth and thereafter, as shown by the steady cell number and the reduced size of colonies (Fig. 1E). The iSML1 iPSCs cultured in the presence of DOX also exhibited a significant reduction in the number of replicating cells, as evidenced by the decreased number of EdU-positive cells, although no differences were observed between cells grown in DOX for 7 or 14 days (Fig. 1F). No reduced viability or replication was observed in naïve parental iPSC cultured in DOX (Fig. S1A,B). Notably, re-expression of the wild-type RNAi-resistant SMARCAL1 in iSML1 iPSCs through an inducible allele-switch approach (see the schematic in Fig. S1C) largely reverted the reduced EdU-incorporation (Fig. S1C,D). Reduced proliferation and number of replicating cells were also observed in normal human primary fibroblasts expressing the inducible shSMARCAL1 construct (Fig. S2A-C), suggesting that the phenotype is independent of the cell cycle type and not specific to iPSCs.

Collectively, these results indicate that inducible long-term depletion of SMARCAL1 in iPSCs is achievable. They also demonstrate that depletion of SMARCAL1, a condition mimicking the severe phenotype of SIOD cells, is associated specifically with a time-dependent reduction in cell proliferation.

### Depletion of SMARCAL1 induces DNA damage and checkpoint activation in iSML1 iPSCs

**Fig. 2.**
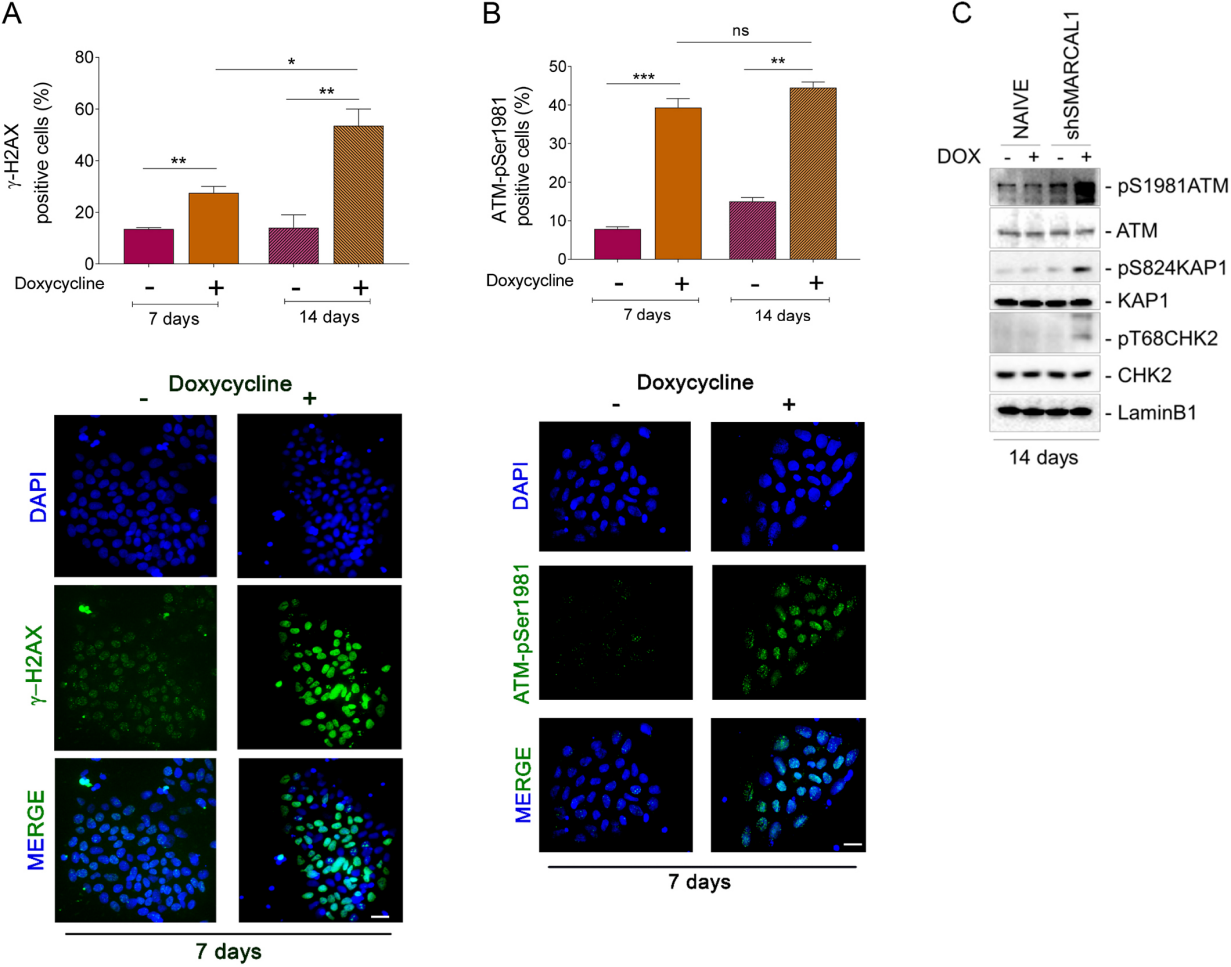
**Depletion of SMARCAL1 induces DNA damage and checkpoint activation in iSML1 iPSCs.** (A,B) The iSML1 iPSCs were cultured for 7 and 14 days in the presence of doxycycline (DOX) to induce SMARCAL1 downregulation and then immunostained. The graphs (top) show quantification of the number of γ-H2AX-positive cells (A) or ATM-pSer1981-positive cells (B). Representative images from triplicate experiments are shown (bottom). (C) Immunoblot detection of the indicated DDR proteins in iSML1 iPSCs after 14 days of continuous treatment with DOX. Lamin B1 was used as the loading control. Data are mean±s.e.m. from three independent experiments. *P≤0.05, **P≤0.01, ***P≤0.001 (two-way ANOVA test). ns, not significant. Scale bars: 10 µm.

**Fig. 3.**
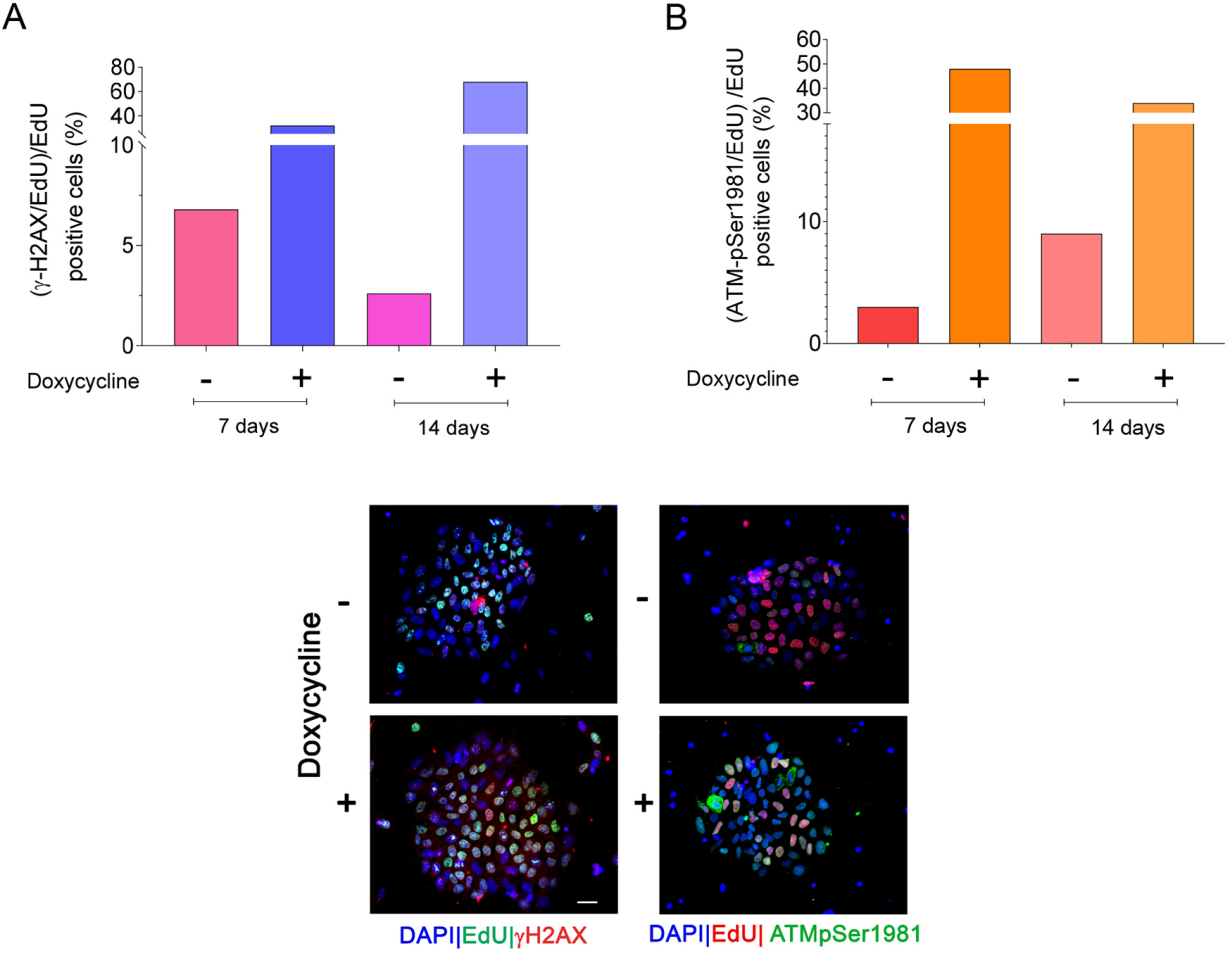
**Enhanced phosphorylation of H2AX and ATM** **is related** **to** **S-phase.** (A,B) Analysis of the level of DNA damage in iSML1 iPSCs cells during replication, at either 7 or 14 days. Cells were cultured in doxycycline+ medium as indicated and S-phase cells labelled with EdU for 30 min before sampling. The graphs report the number of γ-H2AX-positive cells (A) or the number of ATM-pSer1981-positive cells (B) in the S-phase population. Data are means, from biological duplicates. Standard errors are not depicted and are <15% of means. Representative images from 14 days are shown below the graphs; right column, ATM activation; left column, H2AX phosphorylation. Scale bar: 10 µm.

Transformed or cancer-derived SMARCAL1-depleted cells are characterized by elevated levels of DNA damage (Bansbach et al., 2010; Ciccia et al., 2009; Couch et al., 2013). As inducible SMARCAL1 downregulation hampers proliferation in iPSCs (Fig. 1), we analyzed whether this phenotype could correlate with enhanced DNA damage. To this end, we performed single cell immunofluorescence analyses on the presence of two acknowledged markers of DNA damage and checkpoint activation, phosphorylated H2A.X variant histone (γ-H2AX) and ATM (ATM-pSer1981). Depletion of SMARCAL1 by continuous cell growth in DOX resulted in a significant increase in the number of γ-H2AX-positive cells over time, which was otherwise not observed in cells cultured in the absence of DOX or in naïve parental iPSCs cultured in the presence of DOX (Fig. 2A; Fig. S3A,C). Consistent with γ-H2AX data, depletion of SMARCAL1 also triggered ATM activation, as visualized by enhanced Ser1981 phosphorylation (Fig. 2B), an event associated with DNA damage and checkpoint activation. In contrast with γ-H2AX accumulation, the presence of ATM-pSer1981-positive cells was constant between 7 and 14 days of culture in DOX, whereas it showed a small increase over time in iSML1 iPSCs growing in the absence of DOX (Fig. 2B). As shown for γ-H2AX accumulation, simply growing parental iPSCs in DOX did not increase the level of ATM activation (Fig. S3B,C). Increased activation of the DDR in the iSML1 iPSCs was further assessed by western blotting using phosphospecific antibodies for ATM and two of its downstream effectors, CHK2 (CHEK2) and KAP1 (TRIM28). Western blotting analysis confirmed that a 14 day-long depletion of SMARCAL1 activates the DDR and showed that long-term culturing in 1 µg/ml DOX does not, per se, stimulate phosphorylation of the DDR factors (Fig. 2C). To assess whether increased activation was related to loss of SMARCAL1, we evaluated the presence of DNA damage and DDR activation after re-introduction of the wild-type SMARCAL1. Notably, expression of the RNAi-resistant wild-type SMARCAL1 in the iSML1 iPSCs significantly reduced accumulation of γ-H2AX and pATM foci (Fig. S4A,B). Although at a lesser extent than in iPSCs, increased γ-H2AX and ATM-pSer1981 immunofluorescence was also detected in normal human primary fibroblasts after inducible depletion of SMARCAL1 for population doublings corresponding to 14 days after shSMARCAL1 induction (Fig. S5A,B). Consistent with the role of SMARCAL1 as replication caretaker (Bansbach et al., 2009; Ciccia et al., 2009), the large majority of iSML1 iPSCs staining positive for γ-H2AX and pATM foci were from S-phase, although a foci-positive staining was also observed outside S-phase (Fig. 3A,B).

These results indicate that continuous cell proliferation with reduced levels of SMARCAL1 leads to DNA damage accumulation and activation of proteins involved in the DDR. This phenotype is directly related to loss of SMARCAL1 and cell-type independent, but more striking in iPSCs than in primary fibroblasts.

### Depletion of SMARCAL1 in iPSCs resulted in reduced fork speed and defective replication

**Fig. 4.**
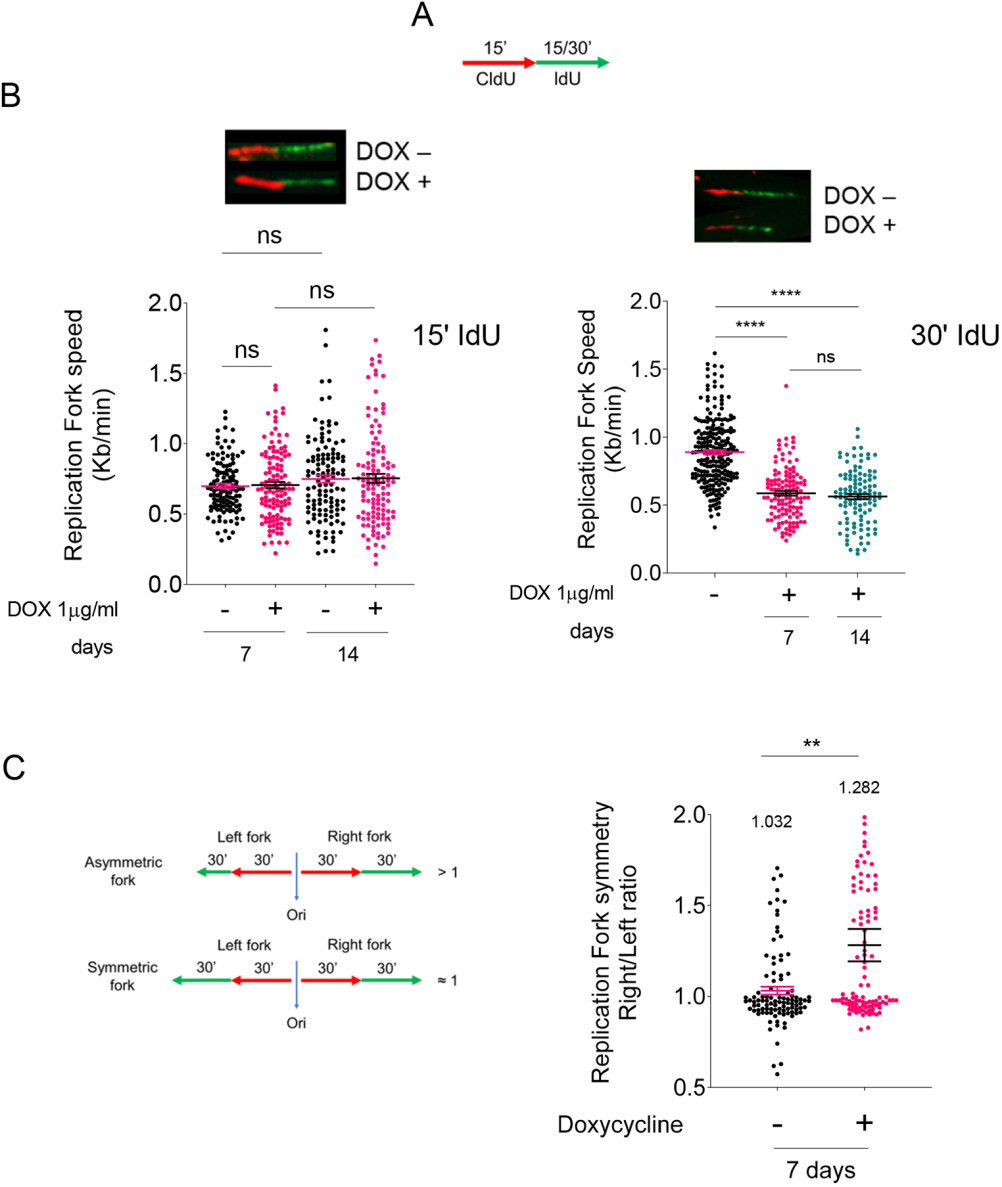
**Analysis of DNA replication in SMARCAL1-depleted iPSCs.** (A) Schematic of the labelling strategy. Cells were labelled by two consecutive pulses of the indicated halogenated nucleotides. (B) The length of the IdU tracts (green; top) was evaluated in at least 150 fibres after 15 min (left) and 30 min (right) and was converted into fork speed values. The graphs (bottom) show scatter plots of single fork speed from iSML1 iPSCs treated as indicated. ****P<0.0001 (Mann–Whitney test). (C) Analyses of the fork symmetry parameter evaluated as indicated in the schematic (left). The graph (right) shows the left/right fork value for each bi-directional fork (*n*=100 from two biological replicates; values on top represent mean ratio for each population). **P≤0.01 (two-way ANOVA test). Data are mean±s.e.m. ns, not significant.

Having demonstrated that depletion of SMARCAL1 reduces proliferation of iPSCs and increases DNA damage, we tested whether it also affected DNA replication dynamics. To this end, we performed single-molecule replication assays using dual-labelling with halogenated thymidine analogues and DNA fibres (Leuzzi et al., 2016). Active replication forks were labelled with two consecutive 5-chloro-2′-deoxyuridine (CldU) and 5-iodo-2′-deoxyuridine (IdU) pulses of 15 min (Fig. 4A). To more accurately evaluate the presence of fork obstacles accumulating with time, we also used a longer IdU pulse (González Besteiro et al., 2019). Analysis of IdU track length in dual-labelled fibres showed that loss of SMARCAL1 did not reduce fork speed in iPSCs at 15 min, although it significantly decreased the length of IdU tracks at 30 min (Fig. 4B). Thus, we analyzed the fork symmetry, another parameter linked to the presence of stalled forks (Técher et al., 2013), at 30 min of IdU labelling (Fig. 4C). Notably, downregulation of SMARCAL1 resulted in an increasing number of asymmetric bidirectional forks, as evidenced by a right/left fork ratio higher than 1, where the right fork is showing the reduced length (Fig. 4C).

Collectively, these results indicate that downregulation of SMARCAL1 in iPSCs affects progression of DNA replication with time, possibly inducing delay or stalling of a subset of ongoing replication forks.

### Preventing replication-transcription conflicts reduces DNA damage and replication defects in SMARCAL1-depleted iPSCs

**Fig. 5.**
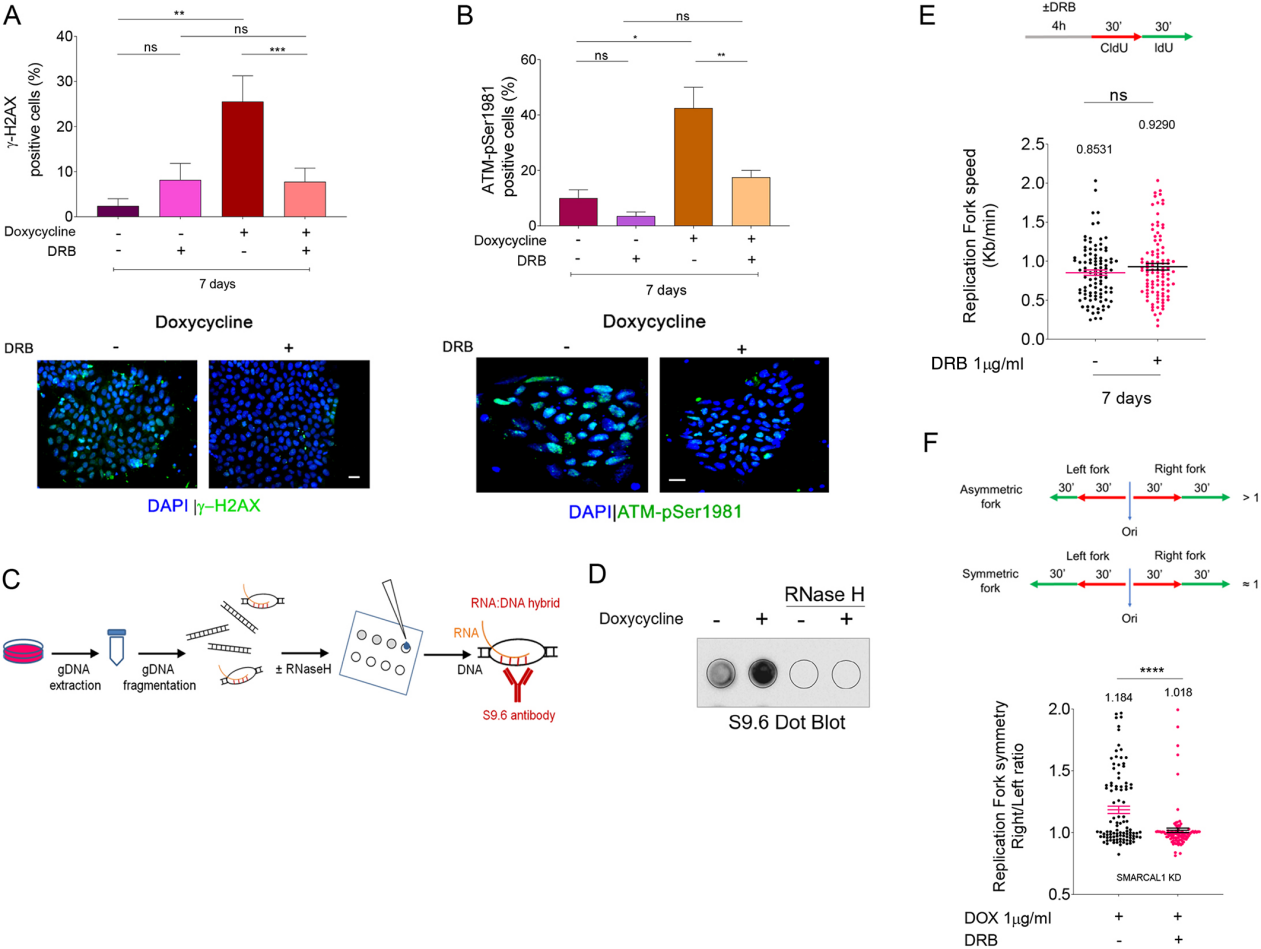
**Preventing replication-transcription conflicts** **reduces DNA damage and DDR in SMARCAL1-depleted iPSCs.** (A,B) Analysis of accumulation of DNA damage in iSML1 iPSCs after SMARCAL1 downregulation. Four hours before sampling, DRB was added in the indicated samples at 50 µM. The graphs show the percentage of γ-H2AX-positive cells (A) or ATM-pSer1981-positive cells (B). Representative images are shown (bottom). Data are mean±s.e.m from three independent experiments. *P<0.05, **P≤0.01, ***P≤0.001 (two-way ANOVA test). (C) Experimental scheme for the detection of R-loops by dot blot in genomic DNA (gDNA). (D) Analysis of R-loop accumulation by dot blotting. Genomic DNA was isolated from iPSCs, treated or not with doxycycline (DOX) to induce SMARCAL1 silencing, and then randomly fragmented before being spotted onto a nitrocellulose membrane. The control membrane was probed with anti-RNA-DNA hybrid S9.6 monoclonal antibody. Treatment with RNase H was used as a negative control. (E) Schematic of the labelling strategy to detect replication fork progression (top). Cells were labelled by two consecutive pulses of 30 min with the indicated halogenated nucleotides. DRB was added, where indicated, 4 h before labelling. The graph (bottom) shows a scatter plot of single fork speed from iSML1 iPSCs treated as indicated. (F) The length of the IdU tracts (green; top) was evaluated in at least 150 fibres. Ori, origin of replication. The graph (bottom) shows a scatter plot of the ratio between the right verses left replication tract (green) from bidirectional forks as shown in the scheme on top. Values are from iSML1 iPSCs treated as indicated. Data are mean±s.e.m. ****P<0.0001 (Mann–Whitney test). ns, not significant. Scale bars: 10 µm.

Embryonic stem cells (ESCs) are characterized by reduced G1-phase (White and Dalton, 2005), a condition reminiscent of cells with activated oncogenes and correlated with enhanced frequency of replication-transcription conflicts (Macheret and Halazonetis, 2018). Hence, we tested whether increased DNA damage observed in iSML1 iPSCs correlated with unresolved replication-transcription conflicts. To this end, we grew iSML1 iPSCs in DOX for 7 days and exposed cells to 5,6-dichloro-1-ß-d-ribofurosylbenzimidazole (DRB) in the last 4 h before performing anti-γ-H2AX immunofluorescence. DRB is a transcription inhibitor widely used to prevent replication-transcription conflicts without affecting, in the short-term, proliferation (Salas-Armenteros et al., 2017), and reduction of γ-H2AX levels in cells treated with DRB indicates that DNA damage originates from replication-transcription interference (Marabitti et al., 2019). Downregulation of SMARCAL1 in iSML1 iPSCs elevated the rate of DNA transcription as evaluated by 5-ethynyl-uridine (EU) incorporation and click-it assay; however, 4 h of DRB treatment similarly suppressed DNA transcription irrespective of DOX (Fig. S6). Treatment with DRB did not significantly affect the presence of γ-H2AX-positive cells in iSML1 iPSCs without DOX; however, it substantially reduced their number in the presence of DOX (i.e. without SMARCAL1) (Fig. 5A). Consistent with the γ-H2AX data, DRB treatment also reduced ATM activation associated with SMARCAL1-depletion (Fig. 5B).

An increased number of replication-transcription conflicts can be associated with enhanced accumulation of R-loops (Hamperl et al., 2017; Lang et al., 2017). To test whether R-loops accumulated after SMARCAL1 downregulation in iPSCs, we purified genomic DNA from cells treated with DOX for 7 days or not treated with DOX and performed dot blot assays to detect R-loops using the S9.6 anti-RNA-DNA hybrids antibody (Boguslawski et al., 1986; Hamperl et al., 2017) (Fig. 5C). As shown in Fig. 5D, cells depleted of SMARCAL1 had substantially elevated levels of genomic R-loops, suggesting that SMARCAL1 contributes to their prevention or resolution.

Accumulation of R-loops and replication-transcription conflicts may underlie DNA replication defects. Thus, we evaluated replication fork rate in iSML1 iPSCs cultured in DOX for 14 days and treated or not treated with DRB for the last 4 h (Fig. 5E). Interestingly, fork speed was unaffected by DRB treatment in SMARCAL1-depleted cells (+DOX), although the fork symmetry was recovered (Fig. 5F).

Therefore, loss of SMARCAL1 accumulates R-loops in iPSCs. Furthermore, our results strongly suggest that DNA damage and replication fork stalling, but not the fork slowing phenotype, depend on accumulation of replication-transcription conflicts.

### Increased levels of replication-dependent DNA damage and checkpoint activation of SMARCAL1-depleted iPSCs persist upon their differentiation

We demonstrated that sustained proliferation in the absence of SMARCAL1 induces the accumulation of replication defects and DNA damage in iSML1 iPSCs. As SIOD affects different cell lineages, we assessed whether the presence of such DNA damage would persist also after spontaneous pluri-lineage differentiation of iPSCs.

**Fig. 6.**
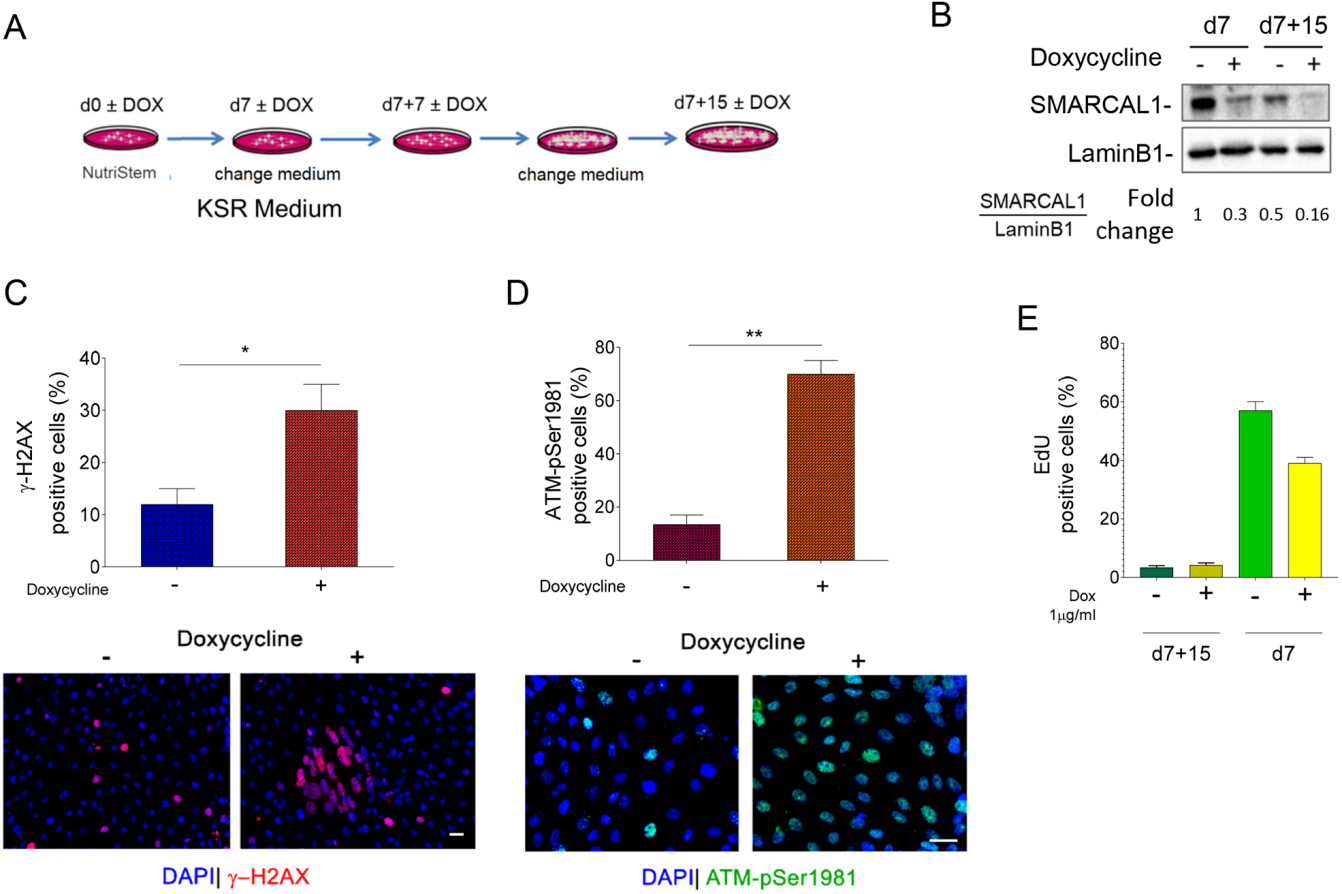
**Increased levels of replication-dependent DNA damage and DDR activation persist in SMARCAL1-depleted iPSCs upon their differentiation.** (A) Schematic of the early differentiation protocol of iSML1 iPSCs, treated with or without doxycycline (DOX) as indicated. (B) Western blot analysis of levels of SMARCAL1 depletion after 7 and 15 days from spontaneous multi-lineage differentiation. Lamin B1 was used as loading control. (C,D) Analysis of DNA damage accumulation or DDR activation in iSML1 iPSCs after spontaneous multi-lineage differentiation. The graphs (top) show the percentage of γ-H2AX-positive nuclei (C) or ATM-pSer1981-positive nuclei (D) for each endpoint. Representative images are shown (bottom). (E) Analysis of replicating cells in SMARCAL1-depleted iPSCs. EdU labelling (30 min) was used to mark replicating cells. The graph shows the percentage of EdU-positive cells after 15 days of early differentiation treated or not with DOX. As reference, the values of EdU-positive cells in respect to the corresponding undifferentiated iSML1 iPSCs are included (d7). Data are mean±s.e.m. from three independent experiments. *P<0.05, **P≤0.01 (two-way ANOVA test). Scale bars: 10 µm.

To this end, iSML1 iPSCs were grown for 7 days in the presence of DOX before switching from pluripotency maintenance medium to differentiation conditions (Lenzi et al., 2015) (Fig. 6A). As shown in Fig. 6B, SMARCAL1 knockdown was stable even in differentiated cells. Consistent with its main function as replication caretaker, the relative amount of SMARCAL1 in iSML1 iPSCs cultured without DOX declined during differentiation. Next, we analyzed the presence of DNA damage using anti-γ-H2AX immunofluorescence in the population of differentiated iSML1 iPSCs. The number of γ-H2AX-positive cells was very limited in the population cultured in the absence of DOX, whereas it was much more elevated in cells with DOX (Fig. 6C). Interestingly, immunofluorescence analysis of ATM activation revealed a much greater difference between cells cultured in the absence or presence of DOX (Fig. 6D). Irrespective of the SMARCAL1 downregulation, the percentage of replicating cells, evaluated using a 5-ethynyl-2′-deoxyuridine (EdU) incorporation assay, was very low and similar at the end of the 15-days differentiation protocol (Fig. 6E). Thus, differences in the number of cells staining positive for the DNA damage markers are unlikely to be related to an excess of undifferentiated replicating cells in the iPSCs growing in the presence of DOX.

**Fig. 7.**
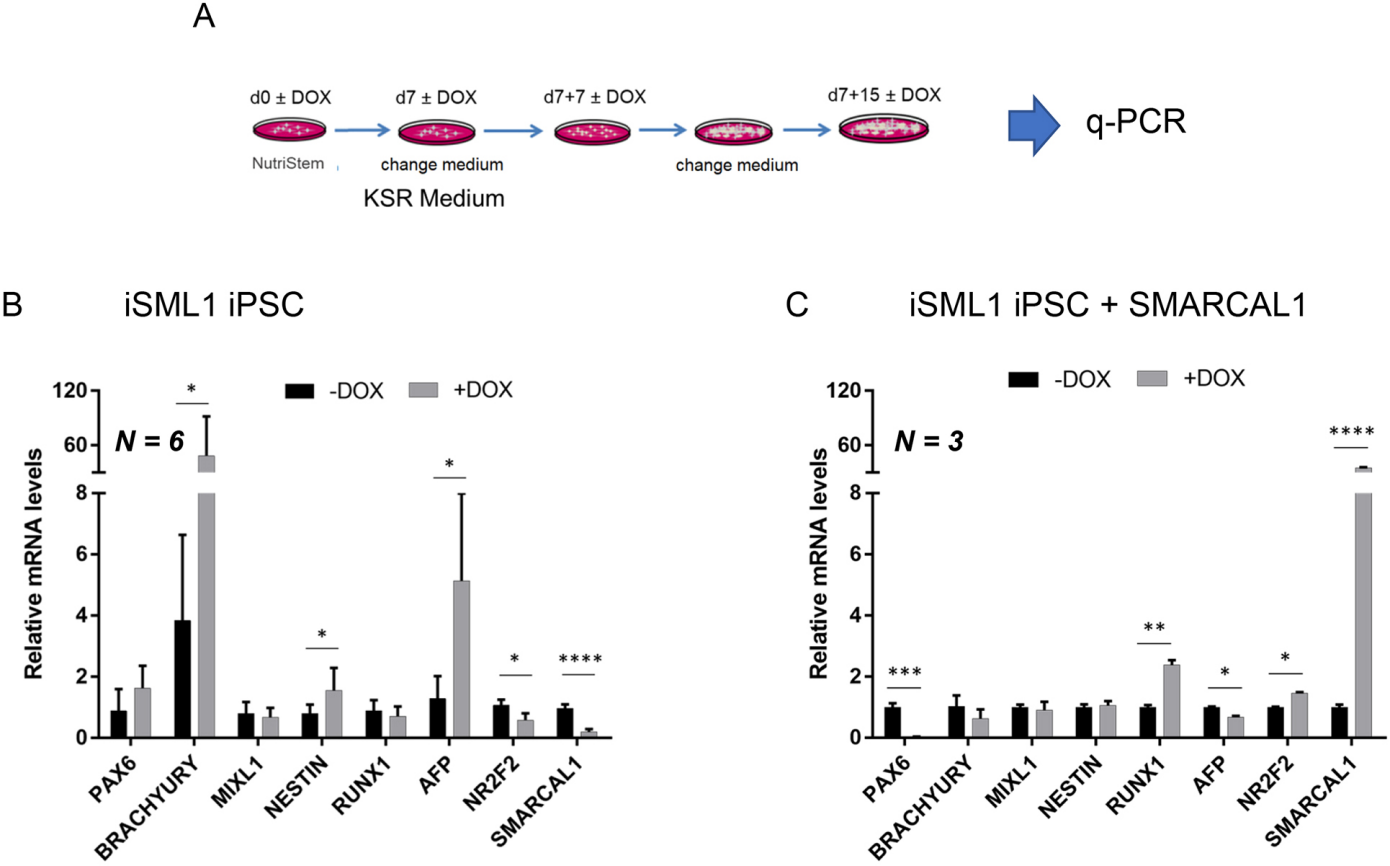
**Alteration of SMARCAL1 affects expression of multi-lineage marker genes after differentiation of iPSCs.** (A) Schematic of the early differentiation protocol of iSML1 iPSCs. Gene expression was evaluated using q-PCR. (B) Comparative analysis by q-PCR of the expression of the indicated early differentiation markers in iSML1 iPSCs. Relative gene expression represents data normalized to *ATP5O* and expressed relative to untreated iSML1 iPSC (DOX–) (*n*=6). (C) Comparative analysis by qRT-PCR of the expression of the indicated early differentiation markers in iSML1 iPSCs induced to express RNAi-resistant wild-type SMARCAL1 in combination with shSMARCAL1. Relative gene expression represents data normalized to *ATP5O* and expressed relative to untreated iSML1 iPSCs (DOX–) (*n*=3). Data are mean±s.d. *P<0.05, **P<0.01; ***P<0.001; ****P<0.0001 (multiple *t*-test analysis).

Our data indicate that chronic depletion of SMARCAL1 in iPSCs stimulates the accumulation of persistent DNA damage and long-term DDR activation, both replication-dependent events. This is also seen in differentiated cells, in which replication is barely detectable. To determine whether such persistent DDR would interfere with pluripotency, we induced spontaneous differentiation into the three germ layers, on a monolayer, and analyzed expression of common marker genes by RT-PCR 15 days after the switch to differentiation medium (KSR medium) (Fig. 7A). Analyses of germ layer-specific genes showed that expression of brachyury (*TBXT*; mesoderm), nestin (*NES*; ectoderm), *AFP* and *NR2F2*, an inhibitor of *OCT4* expressed early during human iPSC differentiation (Rosa and Brivanlou, 2011), was altered in cells depleted of SMARCAL1 (Fig. 7B). These changes in gene expression are unlikely to be the result of spontaneous differentiation in pluripotency conditions due to DNA damage, as no downregulation of the pluripotency marker was observed in DOX-treated undifferentiated cells (Fig. 1D). Changes in the expression of these genes were not observed in the parental naïve iPSC line cultured in the presence of DOX (Fig. S7). Interestingly, re-expression of wild-type SMARCAL1 in the iSML1 iPSCs reverted the effects of SMARCAL1 knockdown on brachyury, nestin, *AFP* and *NR2F2* (Fig. 7B,C). In these cells, alteration of *PAX6* and *RUNX1* expression could be due to non-physiological levels of *SMARCAL1* (Fig. 7C).

Overall, these results indicate that accumulation of DNA damage and increased DDR caused by loss of SMARCAL1 in undifferentiated cells persist even after differentiation into the progenitors of the three germ-layers, suggesting that the effect of the loss of a replication caretaker may be ‘inherited’ by differentiating cells. Moreover, our data suggest that expression of germ layer-specific genes in iPSC-differentiated cells is affected by altered SMARCAL1 levels.

## DISCUSSION

Here, we demonstrate that conditional knockdown of SMARCAL1 in human iPSCs induces replication-dependent and chronic accumulation of DNA damage triggering the DDR. We also establish that DNA damage accumulation and DDR activation can be maintained in SMARCAL1-deficient iPSCs after differentiation, contributing to the altered expression of a subset of germ layer-specific master genes.

Mutations in *SMARCAL1* underlie SIOD (Boerkoel et al., 2002), however how SMARCAL1 loss-of-function correlates with the disease phenotype is unknown. Depletion of SMARCAL1 in transformed cells has been reported to induce spontaneous DNA damage and proliferation defects, which are accrued by induced replication stress (Bansbach et al., 2009; Ciccia et al., 2009; Couch et al., 2013). Similarly, patient-derived transformed fibroblasts are characterized by high levels of DNA damage (Bansbach et al., 2010). Our inducible SMARCAL1 iPSCs appear to recapitulate all these phenotypes, showing the key cellular features of SMARCAL1 loss: spontaneous DNA damage and replication defects.

Of note, all animal models used to investigate SIOD pathogenesis have failed to fully recapitulate the main disease phenotypes (Baradaran-Heravi et al., 2012; Huang et al., 2010). Inducible PSCs are powerful models for studying pathogenetic mechanisms of diseases (Avior et al., 2016). In this regard, our inducible SMARCAL1 knockdown iPSCs may prove useful for identifying the molecular basis of SIOD, especially early molecular events. Moreover, its allele-switch potential can be exploited to investigate genotype-phenotype correlation vis à vis the reported involvement of additional environmental, genetic and/or epigenetic factors in the penetrance of *SMARCAL1* mutations (Baradaran-Heravi et al., 2012; Morimoto et al., 2016a).

SMARCAL1 is a replication caretaker factor and its loss-of-function leads to perturbed replication forks (Couch et al., 2013). Furthermore, ESCs and iPSCs are characterized by spontaneous replication stress and accumulation of remodelled stalled forks (Ahuja et al., 2016). SMARCAL1 is a crucial fork-remodelling protein (Bétous et al., 2012; Kolinjivadi et al., 2017), and so its function is expected to be more important in iPSCs or in ESCs than in other specialized cell types. Indeed, our data show that both DNA damage and activation of ATM increase over cell generation, and mostly develop from S-phase cells. Consistent with this, the proliferation potential of SMARCAL1 knockdown iPSCs does not decline immediately after depletion, but a significant reduction is observed a week after the induced inactivation of the protein. Such a delay suggests that DNA damage or replication stress needs to reach a threshold to induce proliferation arrest and is consistent with increased activation of ATM over time. Of note, our data indicate that conditional depletion of SMARCAL1 is sufficient to induce DNA damage, ATM activation and reduced proliferation in both primary fibroblasts and iPSCs. A telomeric function of SMARCAL1 has been also shown (Poole et al., 2015); however, the persistence of these phenotypes in iPSCs, which re-express telomerase reverse transcriptase (Takahashi et al., 2007), suggests that they are not specifically related to telomere erosion and supports the presence of a more genome-wide replication stress. Interestingly, persistence of phenotypes in iPSCs also differentiate SMARCAL1 loss from that of WRN, another critical replication caretaker (Franchitto and Pichierri, 2014). Indeed, the proliferation potential of cells from patients with Werner syndrome is rescued after reprogramming (Shimamoto et al., 2014). From this point of view, SMARCAL1-depleted iPSCs behave more like those generated from FA-A cells, which are derived from patients with Fanconi anaemia and retain all the key cellular defects of the syndrome (Liu et al., 2014; Rosselli, 2003).

In PSCs, the most likely source of replication stress is linked to a short G1 phase and increased origin firing (Ahuja et al., 2016; Ryba et al., 2010). A similar mechanism for the generation of replication stress has been put forward following oncogene activation (Macheret and Halazonetis, 2018). Of note, in this case, most of the replication stress would derive from interference between replication and transcription (Macheret and Halazonetis, 2018). Conditional knockdown of SMARCAL1 in iPSCs does trigger a substantial accumulation of R-loops, which are linked to replication-transcription conflicts (Hamperl et al., 2017; Lang et al., 2017). This observation would suggest that SMARCAL1 counteracts accumulation of R-loops and replication-transcription conflicts, evidenced by the rescue of DNA damage and ATM activation by transcription inhibition (Fig. 5).

The recent observation of a non-canonical ATM activation, which is dependent on R-loop accumulation and alternative processing (Tresini et al., 2015), and is increased upon defective replication or mild replication stress (Marabitti et al., 2019), is consistent with our data. Notably, although *Smarcal1* knockout mice do not have any significant proliferation defect, they show a slow-growth phenotype and SIOD-related dysfunctions if treated with α-amanitin (Baradaran-Heravi et al., 2012). As α-amanitin interferes with the elongation phase of transcription and not with its initiation, as occurs with DRB, it is possible that slowing RNA polII increases the chance of replication-transcription conflicts in *Smarcal1* knockout mouse embryonic fibroblasts, resulting in a proliferation defect as observed in our iPSC model.

Interestingly, DNA damage and ATM activation caused by replication-transcription interference in iPSCs depleted of SMARCAL1 also persist after spontaneous differentiation in cells of the three germ layers. Thus, a replication-dependent phenotype appears to be inherited in differentiated cells. Most importantly, loss of SMARCAL1 affects expression of a subset of germ layer-specific master genes, which can be rescued through inhibition of DDR signalling. The pathogenetic mechanisms responsible for SIOD are still elusive; however, SMARCAL1 deficiency has been reported to pathologically modulate gene expression (Baradaran-Heravi et al., 2012; Morimoto et al., 2015, 2016a,b; Sanyal et al., 2015). An intriguing possibility is that loss of SMARCAL1 function indirectly influences gene expression through increased levels of replication stress, as has been suggested for loss of WRN or FANCJ helicase (BRIP1) (Khurana and Oberdoerffer, 2015; Papadopoulou et al., 2015; Schiavone et al., 2014). Accumulation or persistence of R-loops could be involved in this mechanism, suggesting the need to assess whether R-loops preferentially accumulate at affected genes. Of note, both ATR- and ATM-dependent signalling have been found to be dysfunctional in cells lacking SMARCAL1, especially following induction of DSBs by doxorubicin treatment (Patne et al., 2017; Sethy et al., 2018). We, and others, have found higher activation of ATM in the absence of SMARCAL1 under unperturbed cell growth or upon perturbed replication (Couch et al., 2013). Possibly, the function of SMARCAL1 as promoter of ATM and ATR transcription, or as a regulator of factors involved in the biosynthesis of long non-coding RNA, is especially important in response to DSBs and not upon replication stress.

One of the germ layer master genes showing increased expression in SMARCAL1 knockdown iPSCs is brachyury. Notably, expression of brachyury has been found to be elevated in cordomas and correlates with increased cellular proliferation in the bone (Miettinen et al., 2015), hence providing a possible link to osseous dysplasia, which is one of the clinical phenotypes of SIOD (Clewing et al., 2007).

Altogether, our work indicates that conditional downregulation of SMARCAL1 in iPSCs recapitulates phenotypes observed in specialized cells following SMARCAL1 depletion. Most importantly, our study demonstrates that loss of SMARCAL1 induces the accumulation of DNA damage and ATM activation in iPSCs through replication stress correlated with replication-transcription conflicts. As mutations in SMARCAL1 cause the multisystemic genetic disease SIOD (Boerkoel et al., 2002) and complete loss of function of SMARCAL1 correlates with the severest form of this condition (Elizondo et al., 2009), our conditional knockdown of SMARCAL1 in iPSCs may represent a powerful model for studying SIOD pathogenetic mechanisms and complement other model systems in recapitulating the disease.

## MATERIALS AND METHODS

### Human iPSC culture, infection and differentiation

Human iPSCs used in this study belong to the WT I line described in Lenzi et al. (2015), where they were authenticated (Lenzi et al., 2015). iSML1 iPSCs were generated by spinfection of iPSCs (passage 13) with the lentiviral vector Tet-ON-shSMARCAL1 at 0.5 of MOI. After 3 days, infected cells were selected with 1 μg/ml puromycin and maintained under selection for 4 days. When indicated, 1 μg/ml doxycycline was added to the medium to induce shSMARCAL1 expression. Established iSML1 iPSCs were maintained in Nutristem-XF (Biological Industries) on plates coated with hESC-qualified Matrigel (BD Biosciences) and passaged every 4-5 days with 1 mg/ml dispase (Gibco). Cells were routinely screened for mycoplasma infection.

For spontaneous pluri-lineage differentiation, 48 h after passaging the culture medium was changed to KSR Medium (DMEM-F12, Sigma-Aldrich; 15% Knockout Serum Replacement, Thermo Fisher Scientific; 1× Glutamax, Thermo Fisher Scientific; 1× Non-Essential Amino acids, Thermo Fisher Scientific; 100 U/ml Penicillin+100 μg/ml Streptomycin, Sigma-Aldrich). Medium was refreshed every other day until the end of differentiation.

### Plasmid construction and transfection

The epB-Bsd-TT-SMARCAL1 construct was generated by inserting the transgene sequence in the enhanced piggyBac transposable vector (Rosa et al., 2014). The *SMARCAL1* open reading frame (ORF) sequence was amplified from the pLVX-IRES-Hyg-PGK-Flag-SMARCAL1 plasmid, which was obtained by PCR cloning of the *SMARCAL1* ORF into the pLVX-IRES-Hyg-PGK vector obtained from Takara-Clontech. The resulting construct was sequence-verified and contains the enhanced piggyBac terminal repeats flanking a constitutive cassette driving the expression of the blasticidin resistance gene fused to the *rtTA* gene and, in the opposite direction, a tetracycline-responsive promoter element driving the conditional expression of the transgene. iPSCs were co-transfected with 4.5 μg of transposable vector and 0.5 μg of the piggyBac transposase using the Neon Transfection System (Life Technologies) as previously described (Lenzi et al., 2015). Selection in 5 μg/ml blasticidin gave rise to a stable/inducible cell line.

### qRT-PCR analysis of differentiation markers

Total RNA was extracted using the Quick RNA MiniPrep kit (Zymo Research) and retrotranscribed using the PrimeScript RT reagent kit (Perfect Real Time). Targets were analyzed by qRT-PCR with SYBR Green Power-UP (Thermo Fisher Scientific) in a 7500 Fast Real Time PCR System (Thermo Fisher Scientific) and calculations performed with the delta delta Ct method. The internal control used was the housekeeping gene *ATP5O* (*ATP5PO*), ubiquitously expressed in human tissues. Primer sequences are reported in Lenzi et al. (2015).

### Growth curve

The cells were dissociated using Accutase (Gibco) and seeded at 2.0×10^4^ cells per plate. Doxycycline induction after 48 h from seeding was considered as the starting point of the growth curve. After trypsinization, cells were counted through electronic counting cells (Bio-Rad) for the following two weeks. After seven days, cells were counted, seeded again at 2.0×10^4^ cells per plate and followed for up to 14 days. The growth curve of the cell cultures was expressed as the number of live cells after Trypan Blue staining as a function of time.

### DNA fibre analysis

Cells were pulse-labelled with 25 μM CldU and then labelled with 250 μM IdU with or without treatment, as reported in the experimental schemes. DNA fibres were prepared and spread out as previously described (Iannascoli et al., 2015). Images were acquired randomly from fields with untangled fibres using an Eclipse 80i Nikon Fluorescence Microscope, equipped with a Video Confocal (ViCo) system. A minimum of 100 individual fibres were analyzed for each experiment, and each experiment was repeated three times.

### Western blot analysis

Western blots were performed using standard methods. The antibodies used are listed below. Blots were developed using Western-bright ECL (Advasta) according to the manufacturer's instructions. Quantification was performed on scanned images of blots using Image Lab software, and values shown on the graphs represent a normalization of the protein content evaluated through lamin B1.

### Antibodies

The primary antibodies used were: anti-SMARCAL1 (#ab154226, 1:1000; Abcam), anti-pCHK2 (#2261, 1:1000; Cell Signaling Technology), anti-CHK2 (#sc5278, 1:1000; Santa-Cruz Biotechnology), anti-pKAP1 (#A300-767A, 1:1000; Bethyl Laboratories), anti-KAP1 (#A300-274A, 1:1000; Bethyl Laboratories), anti p-ATM (#4526, WB 1:800; Cell Signaling Technology), anti-pATM (#05-740, IF 1:300; Millipore), anti-ATM (#NB100-104, 1:1000; Novus Biologicals), anti-pS139H2A.X (#JBW301, 1:1000; Millipore), anti-LaminB1 (#ab16048, 1:20,000; Abcam), rat anti-BrdU (anti-CldU, #ab6326, 1:60; Abcam), mouse anti-BrdU (anti-IdU, #347580, 1:10; Beckton-Dickinson). HRP-conjugated matched secondary antibodies were from Jackson ImmunoResearch and were used at 1:40,000.

### Immunofluorescence

Immunofluorescence microscopy was performed on cells grown on coverslips. Briefly, cells were fixed with 4% paraformaldehyde and permeabilized with 0.4% Triton X-100/PBS. After blocking, coverslips were incubated for 1 h at room temperature with the indicated antibodies. For detection of anti-BrdU, after permeabilization with 0.4% Triton X-100/PBS, cells were denatured in 2.5 N HCl for 45 min at room temperature. Alexa Fluor^®^ 488 conjugated-goat anti mouse and Alexa Fluor^®^ 594 conjugated-goat anti-rabbit secondary antibodies (Life Technologies) were used at 1:200. Nuclei were stained with 4′,6-diamidino-2-phenylindole (DAPI, 1:4000; Serva). Coverslips were observed at 20× objective with the Eclipse 80i Nikon Fluorescence Microscope, equipped with a ViCo system. Images were processed using Photoshop (Adobe) to adjust contrast and brightness. For each time point at least 200 nuclei were examined. Parallel samples incubated with either the appropriate normal serum or only with the secondary antibody confirmed that the observed fluorescence pattern was not attributable to artefacts. Experiments for labelling cellular DNA with EdU or EU were performed by pulse labelling cells with EdU or EU in culture media (10 µM) for 30 min. Detection was performed using Click-iT EdU or EU imaging kits according to the manufacturer's specification (Invitrogen).

### Dot blot analysis

Dot blot analysis was performed according to the protocol previously described (Morales et al., 2016). Genomic DNA was isolated by standard extraction with phenol/chloroform/isoamyl alcohol (pH 8.0) followed by precipitation with 3 M NaOAc and 70% ethanol. Isolated genomic DNA was randomly fragmented overnight at 37°C with a cocktail of restriction enzymes (*Bsr*GI, *Eco*RI, *Hin*dIII, *Xba*I) supplemented with 1 M spermidine. After incubation, digested DNA was cleaned up using phenol/chloroform extraction and standard ethanol precipitation. After sample quantification, 5 μg of digested DNA were incubated with RNase H overnight at 37°C as a negative control. Then, 5 μg of each sample was spotted onto a nitrocellulose membrane, blocked in 5% non-fat dry milk and incubated with the anti-DNA-RNA hybrid [S9.6] antibody (ENH001, 1:1000; Kerafast) overnight at 4°C. Horseradish peroxidase-conjugated goat species-specific secondary antibody (sc-2031; 1:500; Santa Cruz Biotechnology) was used. Quantification on scanned image of blot was performed using Image Lab software (Bio-Rad).

## Supplementary Material

Supplementary information
