# Supplementary material for: Inducible SMARCAL1 knockdown in iPSC reveals a link between replication stress and altered expression of master differentiation genes
Source: Dis Model Mech. 2019 Oct 17;12(10):dmm039487. doi: 10.1242/dmm.039487 (PMC6826020; doi:10.1242/dmm.039487)
Supplement: Supplementary information [file dmm-12-039487-s1.pdf]

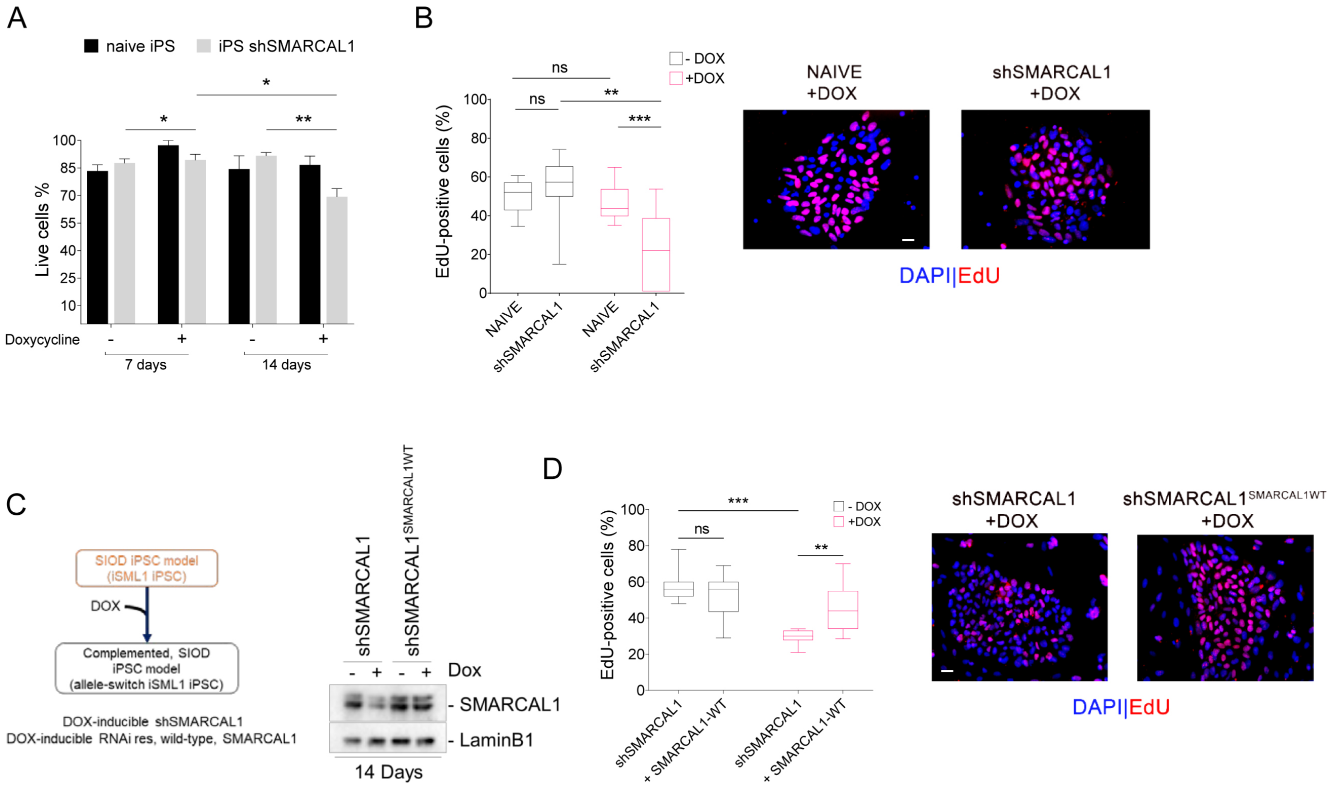

Figure S1. Naïve and wild-type SMARCAL1 iPSCs did not show reduced viability and replicating cells.

(A) Analysis of live cells in naïve and iSML1 iPSCs. Cells were harvested at 7 and 14 days post-treatment with doxycycline and stained with Trypan blue to discriminate live cells. Bar graph shows the percentage of live cells ± SE from three independent experiments. Statistical analysis was performed by ANOVA test, ns= not significant p>0.05; *P ≤ 0.05, **P ≤ 0.01. (B) Analysis of replicating cells in naïve iPSCs. Replicating cells were labelled with EdU for 30min to stain S-phase and the graph plots the percentage of cells positive to EdU after 14 days treatment with doxycycline. Representative images are shown, nuclear DNA was counterstained by DAPI (blue). Statistical analysis was performed by ANOVA test, ns= not significant p>0.05; **P ≤ 0.01, ***P ≤ 0.001. (C) Western blots analysis of SMARCAL1 expression after re-introduction of wild-type SMARCAL1 and 14 days treatment with 0.3µg/ml Doxycycline. Lamin B1 was used as the loading control protein. (D) Analysis of S-phase cells in iSML1 iPSCs re-expressing wild-type SMARCAL1. Cells treated as in (B) were analysed for EdU positivity. The graph shows the percentage of cells positive to EdU signals, as control the values referred to the corresponding iSML1 iPSCs are included. Statistical analysis was performed by ANOVA test, ns= not significant p>0.05; **P ≤ 0.01, ***P ≤ 0.001. Scale bar represents 10µm.

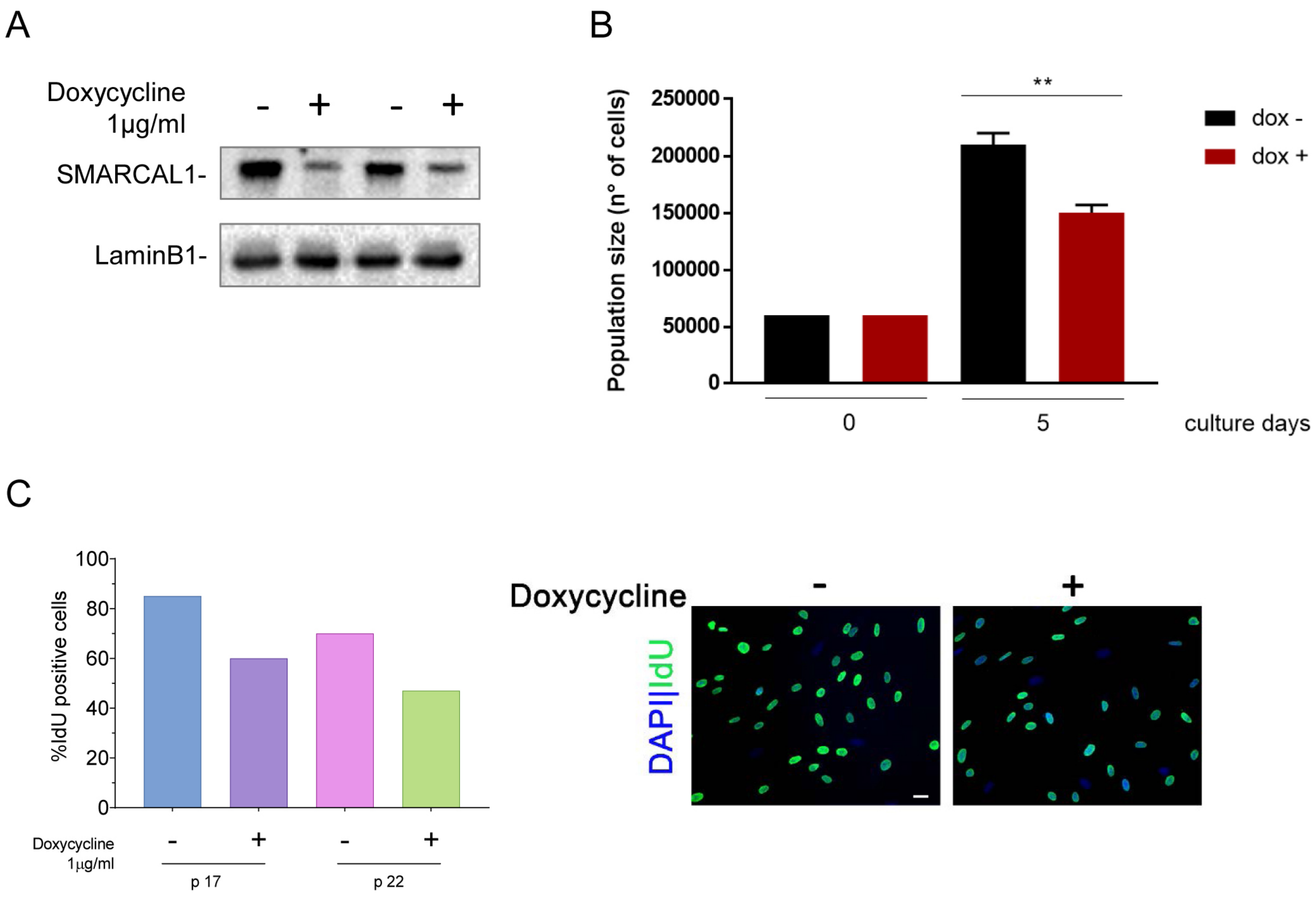

Figure S2. Depletion of SMARCAL1 induced reduced proliferation in normal human primary fibroblasts

A) Western blot showing SMARCAL1 depletion in primary fibroblasts after switching in Dox+ medium. Doxycycline was added a p14 and analysis was performed at p17 and p22. Lamin B1 is used for normalization. B) Evaluation of cell population size for wild-type and shSMARCAL1-induced primary fibroblasts. A starting culture of $6x10^4$ cells was used to plate identical numbers of cells for each cell line and after 5 days in culture the total number of cells was recorded and reported in graph. Data are means±SE from two independent experiments. (**p<0.01; ANOVA test). C) Evaluation of the proliferating population in wild-type and shSMARCAL1-induced primary fibroblasts. Doxycycline was added a p14 and analysis was performed at p17 and p22. Cells were cultured in IdU-containing medium in the last 24h before analysis. Graph shows the number of IdU+. Data are from biological duplicates and are averages. Standard errors are not depicted and are < 15% of means. Representative images of the immunofluorescence experiment are shown. Total DNA is stained with DAPI. Scale bar represents 10µm.

Disease Models & Mechanisms • Supplementary information

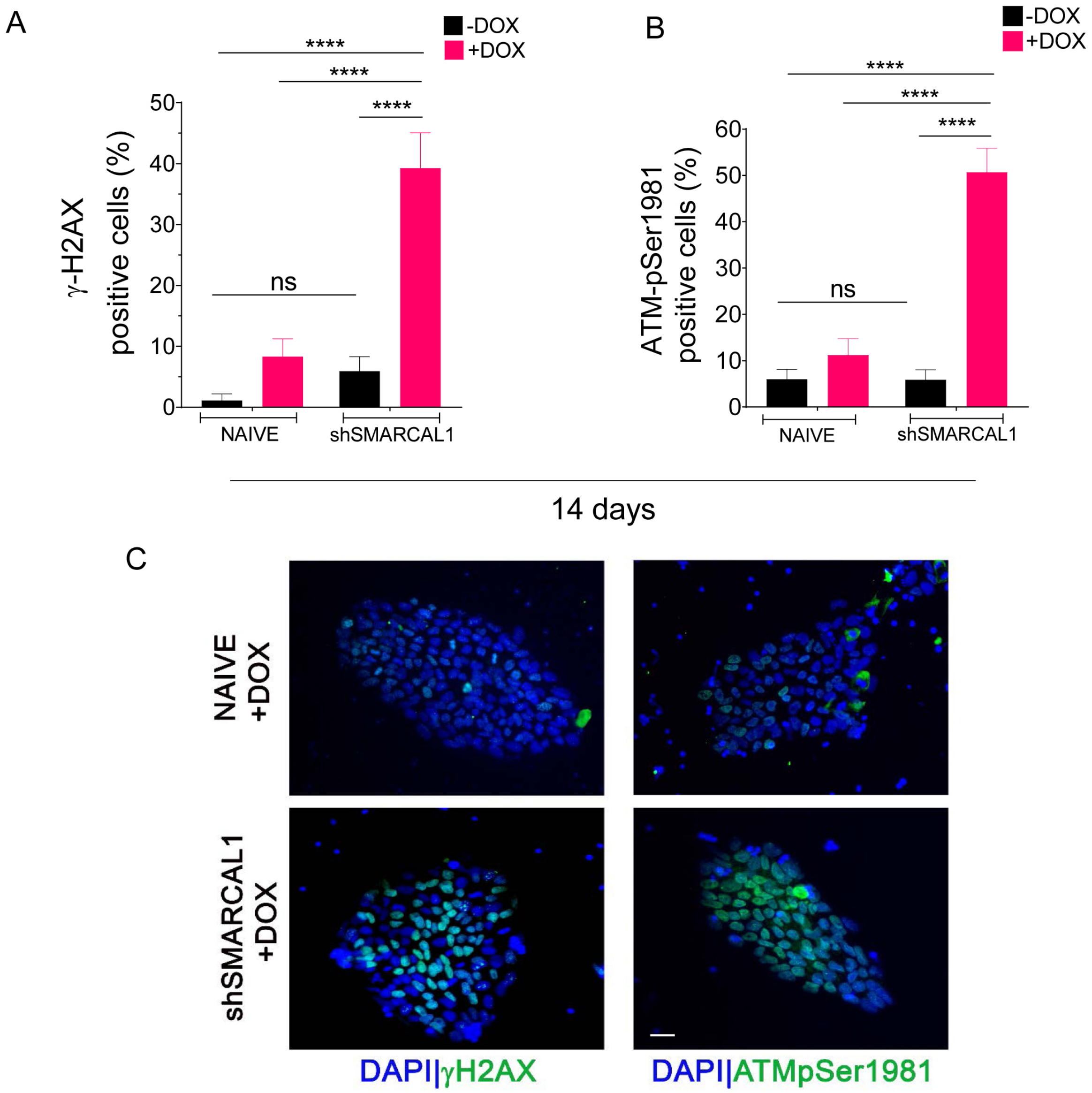

Figure S3. The level of DNA damage and checkpoint activation did not increase in Naïve, parental, iPSCs.

(A-B) Analysis of DNA damage and DDR in parental iPSCs. Doxycycline was added or not for 14 days then cells were immunostained with anti-γ-H2AX or anti-ATM-pS1981 antibody. The graphs represent the analysis of positive cells, compared to shSMARCAL1 iPSCs. Statistical analysis was performed by ANOVA test, ns= not significant, ****P ≤ 0.0001.(C) Panel shows representative images of fluorescence fields from cells stained with anti-γ-H2AX or anti-ATM-pS1981 antibody (green) are provided. Total nuclear DNA was counterstained by DAPI (blue). Scale bar represents 10μm.

Disease Models & Mechanisms • Supplementary information

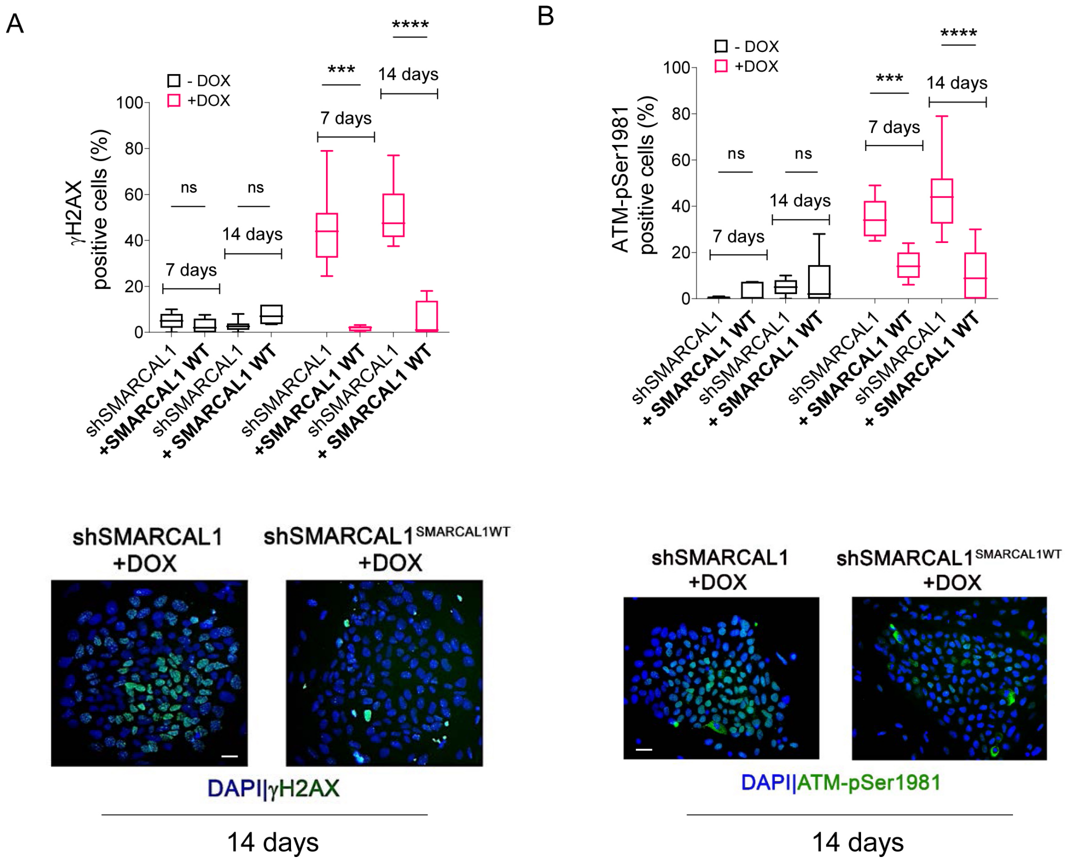

Figure S4. Expression of the RNAi-resistant wild-type SMARCAL1 in the iSML1 iPSC reduced significantly accumulation of γ-H2AX and pATM foci.

(A) Analysis of DNA damage accumulation after treatment or not with Doxycycline for 7 and 14 days. The graphs show the percentage of positive nuclei for each indicated endpoint. Data are presented as mean ± standard error (SE) from two independent experiments. ns=not significant ****P<0.0001; ANOVA test. Representative images from cells stained with anti-γ-H2AX (green) are shown. Total nuclear DNA was counterstained by DAPI (blue). (B) Cells as in (A) were analysed for DDR activation. The graph shows the percentage of positive cells for anti-ATM-pSer1981 antibody. Statistical analysis was performed by ANOVA test, ns= not significant p>0.05; ***P ≤ 0.001, ****P ≤ 0.001. Representative images of positive ATM-pSer1981 cells (green) are provided; nuclei were counterstained with DAPI (blue). Scale bar represents 10μm.

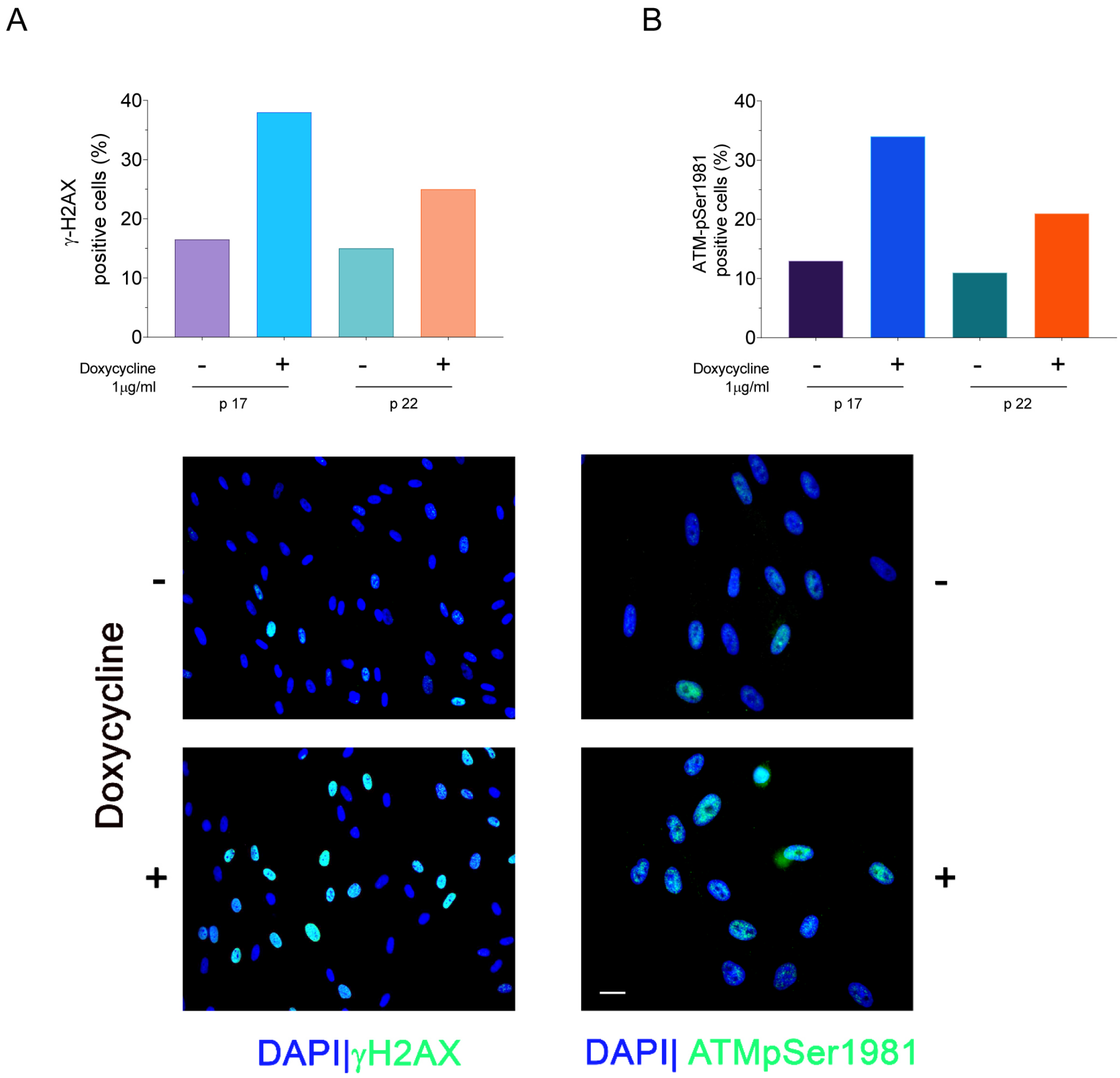

Figure S5. SMARCAL1-silenced primary fibroblast shows increased DNA damage and DDR activation

A-B) Analysis of spontaneous DNA damage and DDR in primary fibroblast depleted for SMARCAL1. Doxycycline was added a p14 and analysis was performed at p17 and p22. Cells were immunostained with anti-γ-H2AX or anti-ATM-pS1981 antibody. The graphs represent the analysis of positive cells after continuous treatment with doxycycline at p17 and p22 (i.e. 7 and 12 days). Representative images of fluorescence fields from cells stained with anti-γ-H2AX or anti-ATM-pS1981 antibody (green) are provided. Total nuclear DNA was counterstained by DAPI (blue). Data are from biological duplicates and are averages. Standard errors are not depicted and are < 15% of means. Scale bar represents 10μm.

Disease Models & Mechanisms • Supplementary information

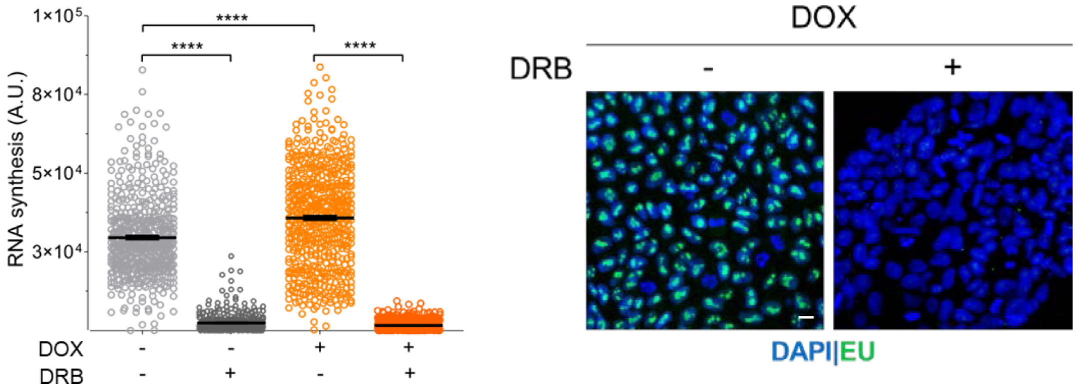

Figure S6. Transcription inhibition prevents RNA synthesis in iSML1 iPSCs.

Analysis of RNA synthesis in iSML1 iPSCs. Cells were labelled with 5'-Ethynil-Uridine (EU) for 1h to stain for active transcription. Four hours before sampling, DRB was added in the indicated samples at 50 µM. Cells were then fixed and subjected to Click-reaction to reveal EU. Dot plot shows the EU fluorescence intensity per cell measured after 14 days treatment with doxycycline. Representative images are shown, nuclear DNA was counterstained by DAPI (blue). Statistical analysis was performed by Mann-Whitney t-test, ****P ≤ 0.0001.

Disease Models & Mechanisms • Supplementary information

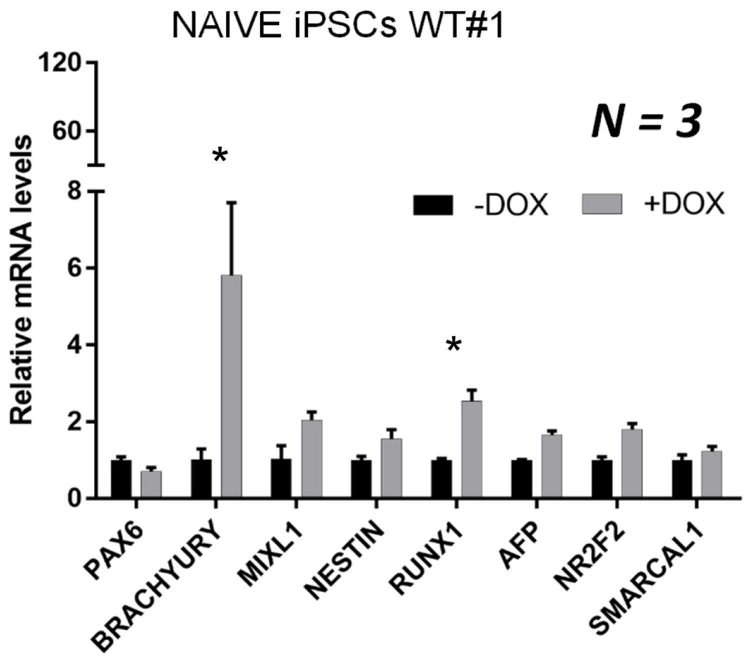

Figure S7. Minimal effects of doxycycline treatment on transcription of genes affected by SMARCAL1 alteration in iPSCs.

Comparative analysis by qRT-PCR of the expression of the early differentiation markers shown in the graph in naïve iPSCs treated as in Figure 7A. Relative gene expression represents data normalized to ATP5O and expressed relative to untreated iPSCs (DOX-) (mean ± S.D.; n = 3. Multiple t-test analysis, *P< 0.05. Scale bars as indicated).

Disease Models & Mechanisms • Supplementary information
